# Spirituality as a coping resource: Towards integration into mental health framework

**DOI:** 10.1371/journal.pmen.0000667

**Published:** 2026-07-24

**Authors:** Sajama Nepali

**Affiliations:** Independent Researcher, Kathmandu, Nepal; PLOS: Public Library of Science, UNITED KINGDOM OF GREAT BRITAIN AND NORTHERN IRELAND

## Abstract

Spirituality has long been recognized as a fundamental dimension of human health and well-being, yet its integration into mental health frameworks remains limited. This essay explores how spirituality is experienced as a coping resource for mental well-being, highlighting mechanisms through which spiritual and religious practices support emotional regulation, resilience, and a sense of purpose. Drawing on theoretical perspectives, empirical research, and lived experiences from diverse cultural contexts including Nepal, it highlights practices such as surrender, mindfulness, detachment, yoga, dietary and hygiene rituals, and selfless service contribute to coping with stress and adversity. The essay also considers the context-dependent and non-linear nature of the spirituality–mental health relationship, potential challenges, including maladaptive interpretations of spiritual beliefs. It underscores the need to balance spirituality with evidence-based mental health care. Recognizing spirituality as a complementary pathway to mental well-being may enhance holistic approaches to mental health and inform interventions that are culturally relevant and personally meaningful.

## What is mental health?

The most widely accepted definition of health is given by the World Health Organization (WHO), which highlights three pillars of well-being: physical, mental, and social. The WHO defines health as *“a state of complete physical, mental, and social well-being and not merely the absence of disease or infirmity”* [1]. Like physical health, mental health is a key component of our health and overall well-being. Traditionally, mental health was understood through the lens of mental illness. However, there has been a shift in understanding mental health to be more than just the absence of mental illness [2]. The WHO further defines mental health as a state of mental well-being that enables people to cope with the stresses of life, realize their abilities, learn and work well, and contribute to their community [3]. This definition emphasizes that mental health is a positive state that enables individuals to function and thrive in their daily lives. The concept of mental well-being further expands this perspective by taking into account positive aspects of mental health such as resilience, the ability to cope with life challenges, the capacity to engage in fulfilling relationships, and having a sense of purpose [4]. From this perspective, mental health can be understood as a holistic state that includes emotional, psychological, and social well-being [5].

Beyond these definitions, mental health exists on a complex continuum and is shaped by a range of interacting individual, familial, community, and structural factors that are experienced differently between individuals [6]. It influences how people think, feel, behave, make decisions, and respond to life’s challenges. Depending on the interplay of protective and risk factors, exposure to adverse circumstances may either foster resilience or increase vulnerability to mental health conditions, which can lead to significant distress, functional impairment, or self-harm. Although many of these conditions can be effectively treated, substantial treatment gaps persist globally due to inadequate mental health resources and services [6].

### Why meaning and transcendence matter in mental well being

Apart from physical and mental health, many modern frameworks argue for a fourth pillar for defining health, i.e., spiritual health. Spiritual health is often defined as having a sense of purpose, meaning, and a connection to something larger than oneself [7]. In 1998, the WHO Executive Board proposed adding spirituality to the official definition. Although this change wasn’t formally written into the constitution due to legal complexities, the proposal marked a major shift in how we view human wellness [8,9].

This evolution in how we consider health mirrors Abraham Maslow’s famous theory on the Hierarchy of Needs. Maslow originally arranged human needs like stairs in a hierarchical order starting from basic human needs (food, shelter, love and respect etc…). He also mentioned that once we feel secure at one level, we naturally move up and finally, we will look up to achieving self-esteem and self-actualization [10]. However, in his final years, Maslow realized that self-actualization wasn’t actually the top of the staircase. He added a further step: ‘Self-transcendence’ that has spiritual touch [11].

Self-transcendence is about moving beyond the individual self and developing a sense of connection with others, humanity and greater purpose. It is the shift from focusing on your own ego to serving a higher purpose or a larger goal. Shortly before his death, Maslow proposed the idea of self-transcendence. He viewed it as a state in which individuals move beyond a narrow, ego-centered identity to a wider perspective characterized by greater autonomy, interconnectedness, and reduced dependence on external approval [11]. Such individuals are less exclusively defined by their cultural or social context, allowing them to identify with broader aspects of humanity and existence.

Just as the WHO and Maslow incorporated the spiritual dimension to their models after years of proposing their initial model, many individuals find themselves inclined toward spirituality as they progress through life. In the beginning, a person much like a young theory is often focused on immediate concerns such as physical health, security, education, relationships and success. For some individuals, particularly those raised within religious or spiritual traditions, questions of meaning, purpose, and transcendence may be central from an early age, while for others such concerns may emerge only later in life, often shaped by life experiences and significant challenges. As people encounter more challenges, they come to realize that physical health, social support and mental stability are not always enough. They begin to seek something deeper. In this way, individuals, much like the models proposed by WHO and Maslow, may naturally progress toward a search for meaning and spiritual fulfillment.

This essay does not argue that spirituality should replace evidence-based mental health care; rather, it highlights spirituality as one of the powerful ways people across cultures make sense of adversity, discover meaning, and experience joy in their lives. It recognizes that spirituality is a complex and context-dependent concept, shaped by cultural, social, and historical factors, and often overlapping with but distinct from religion. The essay examines how individuals in society begin engaging in spiritual practices in response to life challenges and how these practices may support mental well-being by nurturing emotional resilience, inner strength, and connecting to a deeper sense of purpose. It is also aimed at encouraging us to embrace and integrate spirituality for supporting mental well-being.

### Why adversity often leads people towards spirituality

Life is a series of ups and downs. While the up phases are filled with happiness and joy, the down phases often bring stress, anxiety, restlessness and sleeplessness. The acceleration of contemporary society and economic transition has contributed to increasingly demanding work environments characterized by long working hours and pressure to constantly perform and succeed. This has brought about a significant shift in the human experience, which sees more extremes in terms of the highs and lows. These factors can contribute to excessive workloads, job insecurity, and workplace stress, all of which have been associated with poorer mental health outcomes, including anxiety, depression, and other mental health conditions [12]. Indeed, the WHO has estimated that approximately one in four people will be affected by mental health issues at some point in their lives [13].

It is during difficult times that people are more likely to experience mental health challenges such as anxiety and depression. These periods of adversity may also prompt individuals to seek deeper meaning and purpose, leading some to engage more deeply with spirituality and religion [14]. For instance, the interest in religion increased throughout the course of the COVID-19 pandemic [15]. In this context, spiritual practices help many people endure life’s most difficult and uncertain moments and help them to foster emotional resilience.

Despite being practiced widely, spirituality remains underappreciated when it comes to its integration into mainstream mental health discussions [16] and research [17]. It remains a low priority in research agendas, and as a result, its importance is often forgotten or overlooked. Limited attention has also been given to spirituality in mental health related curriculum as well [18]. The absence of a consistent and global spiritual health dimension in mental health frameworks may severely limit our understanding of what effectively supports for many people, leading to interventions for mental well-being that do not meet the needs of many.

### Spirituality Vs religion

Given the role of spirituality and religion in how individuals cope with stress, it is important to clarify what is meant by spirituality and how it relates to, yet differs from, religious belief [19]. Spirituality comes from the Latin word spiritualitas, meaning breath, indicating that it is as vital to us as breathing [20]. It is a personal journey toward finding inner peace, hope, compassion, and kindness and an individual’s relationship with non-material aspect of the universe. It involves the search for meaning and purpose through transcending the self and fostering connection with others, nature, or a supreme being, with or without affiliation to formal religious traditions [21].

The word religion however, is derived from the Latin religare, which means to bind together [20]. Religion is generally viewed as an external, organized system with specific traditions, rituals, and community structures sustained by human institution, ethnic group and culture [17]. Religions have developed throughout history across different societies as communities sought to understand fundamental questions about existence, morality, suffering, and humanity’s relationship with the sacred [22]. Today, religions can be broadly categorized into major world traditions such as Christianity, Islam, Hinduism, Buddhism, Judaism, and numerous Indigenous and folk religions, each with distinct beliefs and practices. Therefore, it is a multidimensional construct encompassing beliefs, behaviors, rituals, and ceremonies practiced privately or publicly, and rooted in established traditions that have developed over time within a community [22]. Although spirituality and religion are conceptually distinct, they often overlap, and both can provide meaning, comfort, and coping resources that contribute to mental well-being.

While these definitions provide a foundation, their meaning shifts across cultures and historical moments. Their understanding has been shaped by broader social and cultural changes, declining participation in religious institutions, and increasing emphasis on personal identity and self-expression [23]. In many societies, including those where religious and spiritual practices remain embedded within communal, familial, and cultural life, the boundaries between religion and spirituality are less sharply drawn [22]. Global survey data suggests that about 84% of the world’s population have reported to follow a religion [19]. Even among those who do not identify with a religion, around 68% still express belief in a higher power or spirituality. These figures highlight that spirituality and religion, though often distinguished conceptually, remain deeply intertwined in lived experience. What is classified as spirituality in one setting may be understood as everyday cultural or religious practice in another. As a result, interpretations of spirituality and religion must account for the social and cultural environments in which these concepts are born and lived.

### Complexity in the spirituality–Mental health relationship

This complexity is reflected in research findings, which show mixed and context-dependent findings. There are numerous studies that have tried to understand the relationship between spirituality and mental well-being, with mixed findings. For instance, Koenig (1998) conceptualizes religion as a multidimensional resource that can provide meaning, social support, and coping strategies, thereby contributing to psychological resilience during stress [24]. However, Koenig also emphasizes that findings are not uniform, and the relationship between religion and mental health varies depending on context, measures of religiosity, and mental health outcomes.

In contrast to this, a study conducted in the UK between 2006–2007 raised an important observation that people identifying as spiritual but not religious reported higher levels of several psychiatric symptoms [25]. The study found increased prevalence of anxiety disorders, phobias, and substance use disorders among the spiritual but not religious group. The findings may reflect the psychological circumstances that lead individuals toward spiritual seeking rather than evidence that spirituality itself as a risk factor. Additionally, the interpretation of these findings also requires attention to the socio-political context in which the categories of religious and spiritual are defined. In contemporary England, where institutional religious participation has declined and individualized forms of meaning-making have gained prominence, identifying as spiritual but not religious may reflect a distinctive social identity rather than a universal form of spirituality [23]. Consequently, the association between spirituality and mental health observed in this study may be partly rooted in the social meanings attached to spirituality in Western and other contexts may not be readily generalizable across cultural contexts. Also, the findings from the cross-sectional nature of the study could not confirm causation.

### What research and lived experience suggest

In 1997, the Mental Health Foundation in the UK led the first national survey designed by people who use mental health services. They found that over half of the survey respondents held spiritual beliefs that played a positive role in their lives [26]. In another study among individuals with mental health conditions in the UK, they expressed spirituality as having a sense of purpose; comfort; grounding; the allowance of expression of personal pain and the development of an inner love and compassion for others [27]. As a qualitative study based on participants’ personal experiences, the findings offer rich insights but may not be representative of all individuals experiencing mental distress and belong to specific UK sample, limiting their generalizability to broader populations and different cultural contexts. While these findings highlight supportive role of spirituality, mental health professionals have suggested that religion and spirituality may conversely increase feelings of guilt through an emphasis on sin, potentially contributing to depression [20]. However, this concern is not supported by most empirical studies. For instance, a review article on the relationship between spirituality and mental health conditions found that the majority of research reported an inverse association between religiosity or spirituality and depressive symptoms (greater belief in religion or spirituality were accompanied by lower levels of depression) [20]. Similar findings were reported by other cross-sectional studies in which a lower level of reported depressive symptoms was associated with religious ritual participation [28] and a belief in a transcendent being [29]. On the spiritual side, individuals who report finding a sense of meaning and purpose are also more likely to see a reduction in internal symptoms like sadness, hopelessness, and extreme fatigue [30]. This sense of purpose may make daily tasks feel more manageable [30]. On the religious side, the external social support found within a faith community provides empathy, and so the feeling of being understood might help to reduce social isolation [24]. The mechanisms underlying this effect may involve the sharing of thoughts and feelings with empathetic others, which provides the interpersonal support and emotional resources necessary to cope with mental health condition such as depression [24].

These findings, however, must be interpreted within their broader cultural and social contexts. The ways in which spirituality, religion, and mental health are understood vary considerably across societies and are shaped by local beliefs, histories, and systems of health care [31,32]. In South Asia, for example, the culture of visiting spiritual and traditional healers still exist and they are often important sources of support for individuals experiencing mental health disorder [33]. Studies from India have shown that mental health conditions are frequently interpreted through spiritual or supernatural frameworks, which influence both explanatory models of illness and help-seeking behaviour [34].

While such interpretations may provide meaning, comfort, and social support, they can also present challenges when mental health conditions are understood solely through the lens of sin, spirit possession, karma, or other supernatural causes [33]. In some cases, these beliefs may reinforce stigma, discourage open discussion of symptoms, or delay access to evidence-based mental health care [35–37]. Many individuals and families navigate both systems simultaneously, seeking support from psychologists and psychiatrists while also consulting spiritual or traditional healers [33]. This dual engagement highlights the need for culturally responsive mental health services that respect people’s spiritual worldviews while promoting timely access to effective clinical care. The strengthening of cultural competence among mental health professionals and fostering collaboration with the trusted community and faith-based resources may help reduce barriers to care while improving service acceptability and engagement.

Furthermore, experiences of spirituality and mental health are not shaped by culture alone. An intersectional perspective highlights how factors such as gender, age, socioeconomic status, ethnicity, religion, and other social identities interact to influence experiences of suffering, coping, and access to support [31,32]. As a result, spirituality may hold different meanings and serve different functions across individuals and communities, underscoring the importance of understanding mental health within its broader social and structural context.

### How people use spirituality and religion to cope

The following sections outlines key spiritual and religious practices that individuals commonly use to cope with stress and adversity. Each subsection highlights the underlying mechanism through which these practices may support emotional regulation, resilience, and overall mental well-being (see Table 1).

**Table 1:** Different kinds of spiritual and religious practices as a coping resource for mental well-being.

Spiritual Dimension / Practice	Description	Mental Health Outcomes
Human Virtues	Cultivation of positive emotions such as hope, love, compassion, gratitude, forgiveness through spiritual teachings [17]	Lower stress; enhanced resilience; improved emotional stability [38]
Act of Surrendering	Entrusting uncontrollable situations to a higher power [17]	Reduced anxiety; greater peace of mind; adaptive coping [16]
Viewing Crisis as a Lesson	Interpreting adversity as meaningful growth, connecting with sense of purpose in life [15]	Enhance positivity, reduce depression and anxiety; improved endurance during hardship [20]
Visiting Sacred Spaces / Spiritual and Religious Leaders	Sharing burdens in temples, churches, mosques with higher power or spiritual and religious leaders [39]	Emotional relief; feel relax
Healthy Eating Motivated by Spirituality	Ritualized consumption of fruits, vegetables, home-cooked meals [40]	Reduced depressive symptoms; improved optimism
Religious Fasting	Temporary dietary restriction framed as spiritual discipline	Improvement in depression and anxiety [41]
Hygiene & Cleaning Rituals	Ritual bathing, decluttering, environmental cleanliness [42]	Mood enhancement; increased emotional comfort; sense of dignity [43]
Yoga	Integrated movement, breath regulation, focused attention	Calmness and relaxation; Reduced anxiety and depression; improved mind-body awareness [44]
Detachment & Mindfulness	Non-attachment to ego and material comparison; present-moment awareness	Reduced envy, anger, resentment; improved social harmony [45]
Selfless Service / Volunteering	Helping others in need	Reduced feeling of helplessness; increase feeling of self-worth [46]

#### Human virtues in spirituality.

Globally, all the spiritual and religious traditions place strong emphasis on human virtues and cultivating emotions such as hope, love, compassion, gratitude and forgiveness [22]. In line with this statement, a review of the literature on religious involvement and spirituality highlights that people having religious belief have reported having feeling of hope, love and compassion [17]. When practiced over time, these attitudes can shape how people interpret adversity and relate to themselves and others, often making distress feel more manageable [17]. While the exact biological pathways are complex and still being studied, a growing body of research suggests that prolonged emotional stress is associated with poorer overall health and reduced psychological resilience [47]. In this sense, spiritual and religious practices that encourage emotional balance may contribute to well-being not by reducing potential stigma around mental illness and curing the illness, but by helping people regulate stress and sustain a more stable inner life [38].

#### Detachment and mindfulness.

Another important contribution of spirituality towards mental well-being is through the principle of detachment and mindfulness. Detachment may reduce ego-driven stress and interpersonal conflict. It helps individual loosen their identification with personal narratives, expectations, or comparisons with others. By encouraging alignment with values beyond material success, detachment may eventually decrease feelings of envy, anger, and resentment, thereby improving interpersonal relationships and social harmony [48]. Mindfulness further supports this process by fostering present-moment awareness and a non-judgmental stance toward thoughts and emotions, allowing individuals to observe internal experiences without excessive reactivity [49]. Together, these practices encourage alignment with values beyond material achievement and external validation, which may reduce negative affective states such as envy, anger, and resentment, thereby supporting healthier interpersonal relationships and social functioning [45]. Overall, detachment and mindfulness as a spiritual practice supports mental well-being by fostering emotional regulation, resilience, acceptance, and inner peace, a key component of mental well-being that complement contemporary mental health frameworks.

#### Act of surrendering.

Many religions frame adversity as purposeful by emphasizing the role of a transcendent force in shaping events that lie beyond individual control [17]. In South Asian context, particularly Nepal, spiritual belief systems often play a role in coping with stress where individuals claim leaving everything into the hands of a higher power or God when they find the situation out of control [33,50]. This is the act of surrendering themselves or placing a burden that is too heavy to carry into the hand of a God. It often gives a sense of peace of mind. It may also stop people from repeatedly dwelling on the more difficult thoughts and emotions. Although arising from different philosophical traditions, this process bears conceptual similarities to acceptance-based approaches such as Acceptance and Commitment Therapy (ACT), which encourage individuals to accept difficult experiences rather than struggle against them, while committing to actions aligned with personal values [51]. To support this finding, a review of literature suggests that religious beliefs help people change how they look at difficult situations or reinterpret events that are seen as uncontrollable, making them feel less stressful [16]. Consistent with this, Peneycad et al. (2024) found that individuals who identified as belonging to religious groups (p < .001) and those who considered themselves spiritual (p = .053) reported greater use of emotional engagement coping strategies compared with atheists [15].

#### Acknowledging crisis as a lesson.

Another spiritual and religious perspective that may support mental well-being involves viewing adversity as an opportunity for learning, or deeper understanding. The crisis is interpreted through broader framework of meaning and purpose, which help individuals reappraise adversity as a challenge rather than threat and serves as a lesson for growth [15]. This kind of mindset acts as a powerful cognitive tool to see positively even in darkest hours and often helps to keep a person’s inner peace intact, providing the mental strength needed through hardship [6]. These concepts are more prominent across Asian regions [52]. Religion offers these kinds of coping resources that might help individuals manage stress by fostering positive emotions, reducing the vulnerability to common mental health conditions, including depression, anxiety, suicidality, or substance misuse [20]. These spiritual and religious coping resources include deeply held beliefs that might help individuals find meaning in adversity and sustain a sense of purpose during difficult life circumstances.

#### Visiting temples and churches.

Visiting sacred spaces such as temples, churches, and mosques may provide emotional comfort and psychological relief for some individuals. The act of visiting sacred spaces, such as temples, church and mosques, to share one’s burdens is often considered relaxing, especially in the context of Asia. Many times, individuals may not be able to share their stress, secrets and fear to their families and friends. They often find comfort in sharing such confidential information and griefs to higher power or God in the form of prayers while visiting temples, churches and mosques. A systematic review on spirituality and trauma recovery suggests that for many people in the United States, religious leaders are often the front-line mental health workers [39]. Instead of going to a doctor or therapist first, many people often turn to their spiritual or religious leaders when they are struggling with trauma. These practices are similar to the process of venting out and releasing the emotional turmoil. By externalizing their internal turmoil in a sacred environment, individuals may let go of their anxieties, often allowing them to leave the temple feeling lighter and relaxed.

#### Healthy eating habits in relation to spirituality.

Spirituality often incorporates embodied practices that may support mental well-being through the interconnectedness of mind, body, and meaning. Many spiritual traditions encourage health-promoting behaviors such as consuming fruits and vegetables, preparing meals mindfully, and engaging in periods of fasting. These practices may contribute to mental well-being not only through their physiological effects but also by providing structure, self-discipline, and a sense of purpose. For example, higher fruit and vegetable consumption has been associated with lower psychological distress and depressive symptoms as well as positive well-being such as optimism and self-efficacy [40]. Similarly, a systematic review of religious fasting, including Ramadan fasting, reported improvements in depressive symptoms and reductions in anxiety in several studies [41]. While biological mechanisms such as reduced inflammation, improved gut-brain communication, and enhanced neurotransmitter function may partly explain these associations [53,54], spiritual practices may also strengthen coping by fostering self-awareness, meaning-making, and a sense of connection. Together, these findings highlight how spirituality might influence mental health through both behavioral and psychosocial pathways.

#### Hygiene and cleaning rituals.

Alongside dietary and fasting practices, many spiritual and religious traditions emphasize maintaining personal hygiene. Rituals such as bathing before visiting a temple, performing prayers, and keeping the home and surroundings clean may provide a sense of dignity, and emotional comfort during periods of stress. These practices carry social and cultural value and are often associated with prestige and shared civic responsibility [42]. Emerging evidences suggests that engagement in cleaning and decluttering has been associated with therapeutic effects, particularly for enhancing mood [43].

#### Yoga.

Yoga is widely recognized as a spiritually rooted, embodied practice that integrates movement, breath regulation, and attention. Many practitioners report experiences of calmness and relaxation through these tools that help them in reducing anxiety. In addition, a growing body of research has documented the positive effects of yoga practice on mental health outcomes, including mindfulness, resilience, emotional regulation, happiness, overall well-being, life satisfaction, self-compassion, and social connectedness [55,56]. Other studies have further demonstrated its therapeutic benefits for a range of psychological and neuropsychiatric conditions, such as depression, anxiety, fatigue, eating disorders, and sleep disturbances [44]. Rather than functioning solely as a treatment modality, yoga may support mental health by enhancing mind–body awareness and adaptive stress regulation.

#### Selfless service.

Lastly, volunteering or selfless service has been embedded in spiritual traditions since ancient times. People who believe in spirituality help others in need by giving them their time and energy. A systematic review investigating the mental health impacts of volunteering revealed that by helping others in need one may enhance a sense of purpose, strengthen self-worth, and increase social connectedness, while fostering feelings of usefulness that can counteract helplessness [46]. The review also stated that while mechanism remain unclear, more rigor designed research is needed to establish a clear association.

In his book Man’s Search for Meaning, Viktor Frankl, a psychiatrist and Holocaust survivor, reflected on how people endured the extreme conditions of Nazi concentration camps [57]. He observed that survival was not determined only by physical strength, but also by whether a person could hold onto some sense of meaning, however fragile. For Frankl, this meaning was often found in relationships, in responsibility toward others, or in the hope of completing unfinished work.

What his account highlights is not a medical formula for survival, but an existential truth about the human mind: when people can locate their suffering within a broader narrative of purpose, they are better able to psychologically endure it. Even in environments designed to strip away dignity, Frankl described how small acts of kindness and moments of connection allowed individuals to experience themselves as more than victims. This insight continues to resonate in mental health today, where helping people rebuild meaning and identity is often as important as reducing symptoms.

## Conclusions

Globally, spirituality has long been part of how human beings make sense of adversity, yet it remains marginal in mainstream mental health discourse. This essay has shown that mental well-being is not only about the absence of symptoms, but about the presence of meaning, connection, and inner stability. From the WHO’s evolving view of health, to Maslow’s idea of self-transcendence, to research on spiritual and religious coping, a consistent pattern emerges: people endure psychological distress more effectively when they can locate their pain within a larger framework of purpose.

Individuals turn to spirituality in moments of fear, loss, and uncertainty, not because it eliminates pain and grief, but because it helps transform it. Whether through prayer, ritual, detachment, surrender, or service, spiritual practices allow people to regulate distress, externalize overwhelming emotions, and restore a sense of dignity and hope. At the same time, spirituality is not without complexity, and spiritual struggle can also deepen distress, reminding us that it must be approached with sensitivity rather than idealization. Also, spiritual beliefs and practices are deeply shaped by culture, any meaningful integration of spirituality into mental health understanding and care requires attention to the specific cultural contexts in which these beliefs are lived and experienced.

Recognizing spirituality as a coping resource does not mean replacing psychological or medical care; rather, it involves acknowledging the full reality of how people live with and survive mental pain. A more humane and effective mental health framework makes room not only for symptoms and treatments, but also for meaning, transcendence, and the deeply human search for something beyond the self. Centering the lived experiences of individuals and communities who already draw on spiritual resources is essential to understanding how such practices function in everyday contexts. Future research should therefore examine how spirituality can be responsibly integrated into culturally sensitive, community-based mental health interventions, supported by dedicated funding that enables interdisciplinary collaboration and sustained engagement with people with lived experiences. Without investment in this area, important dimensions of resilience and recovery risk remaining underexplored and underutilized.
